# Improving Perioperative Outcomes Through Minimally Invasive and Non-invasive Hemodynamic Monitoring Techniques

**DOI:** 10.3389/fmed.2018.00144

**Published:** 2018-05-17

**Authors:** Takashige Yamada, Susana Vacas, Yann Gricourt, Maxime Cannesson

**Affiliations:** ^1^Department of Anesthesiology and Perioperative Medicine, University of California, Los Angeles, Los Angeles, CA, United States; ^2^Department of Anesthesiology, Keio University School of Medicine, Tokyo, Japan; ^3^Departement Anesthesie Réanimation Douleur Urgence, Centre Hospitalaire Universitaire Caremeau, Nimes, France

**Keywords:** hemodynamic monitoring, non-invasive, perioperative complications, outcomes, hemodynamic, blood pressure, perioperative outcomes, monitor

## Abstract

An increasing number of patients require precise intraoperative hemodynamic monitoring due to aging and comorbidities. To prevent undesirable outcomes from intraoperative hypotension or hypoperfusion, appropriate threshold settings are required. These setting can vary widely from patient to patient. Goal-directed therapy techniques allow for flow monitoring as the standard for perioperative fluid management. Based on the concept of personalized medicine, individual assessment and treatment are more advantageous than conventional or uniform interventions. The recent development of minimally and noninvasive monitoring devices make it possible to apply detailed control, tracking, and observation of broad patient populations, all while reducing adverse complications. In this manuscript, we review the monitoring features of each device, together with possible advantages and disadvantages of their use in optimizing patient hemodynamic management.

## Introduction

While medicine is moving toward standardized care, the 2015 Precision Medicine Initiative aimed to understand how a person's genetics, environment, and lifestyle can help determine the best approach to prevent or treat disease. It is now possible to improve patient outcomes by setting individualized hemodynamic parameters according to specific and customized comorbidities or current pathologies. Enhanced Recovery After Surgery (ERAS) protocols recommend individualized intraoperative fluid optimization through integrated hemodynamic monitoring (1).

For patients and healthcare providers, blood pressure (BP) is one of the most important vital signs monitored. The recent development of monitoring technologies allows clinicians to obtain both minimally invasive and continuously non-invasive BP. Hemodynamics describes a patients' BP, cardiac output (CO), and systemic vascular resistance (SVR). Appropriate and precise evaluations of these parameters make it possible to evaluate tissue perfusion. Although the optimal hemodynamic parameters for each patient are undefined, patient outcomes can potentially be improved by applying therapeutic strategies based on hemodynamic information (2). Poor perioperative hemodynamic management of surgical patients can extend beyond cardiovascular complications. Appropriate management can potentially lead to a decrease in neurologic complications, kidney injury, and even mortality.

The goal of this review is to describe the existing scientific scholarship on perioperative hemodynamic monitoring techniques and patient outcomes. We will describe different monitoring techniques as well as the advantages and disadvantages of each device. By using tailored monitoring tools, it is possible to adjust therapeutic decisions for each patient individually and for specific situations.

## Hemodynamic physiology

Blood circulation supplies the necessary nutrients and oxygen to each tissue and collects unnecessary or toxic substances. The proper maintenance of pressure is necessary to distribute enough blood so that the organism can adapt to vigorous activity. Normally, organisms maintain their circulation homeostasis adequately, but surgery, anesthesia, and/or critical illness may disturb this homeostasis. Accurate hemodynamic monitoring is mandatory in these situations, most particularly with vulnerable patients who might not be able to adequately adapt to these unique conditions. Accurate monitoring provides necessary and invaluable information to launch appropriate interventions.

**Figure 1:**
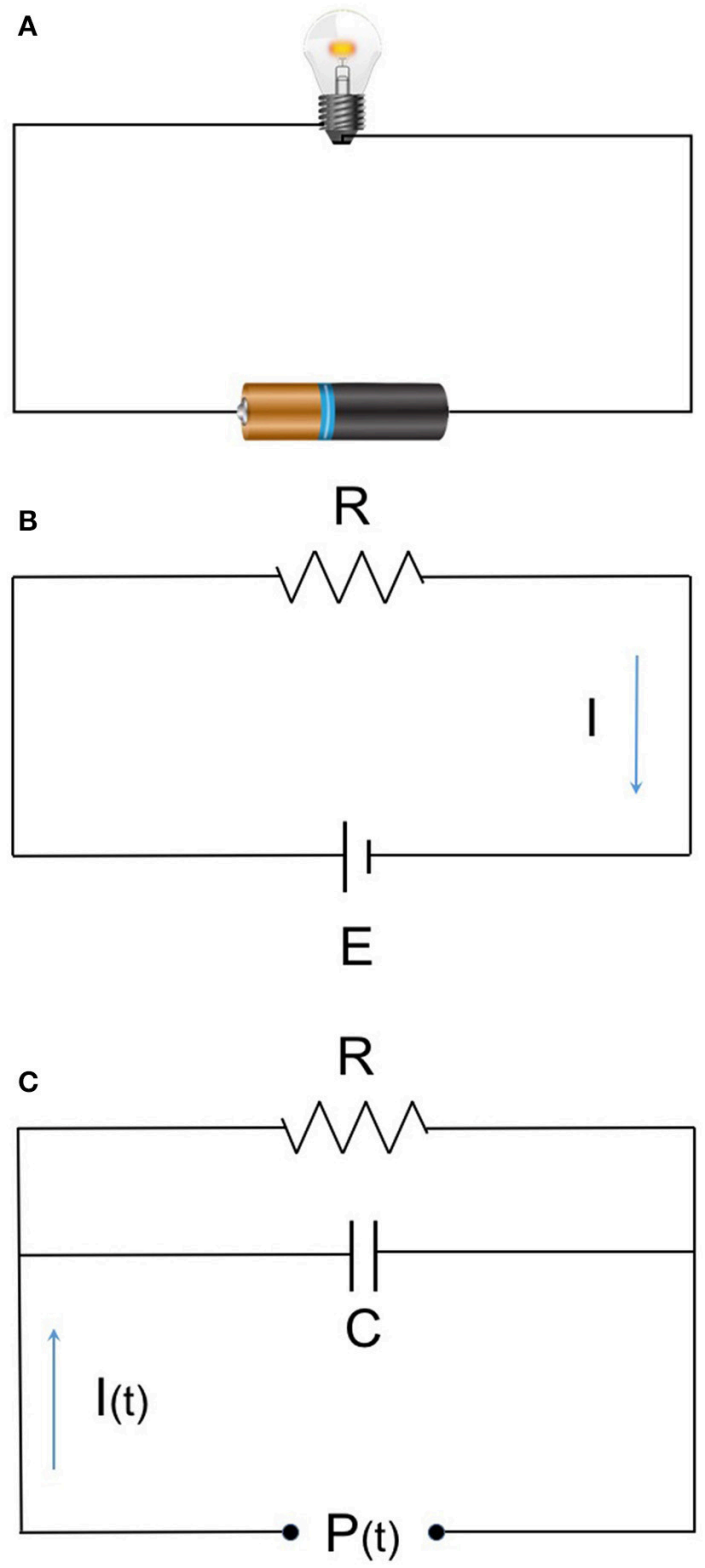
**(A)** Ohm's low and hemodynamic equation. **(B)** E (voltage) = I (current) × R (resistance) (MAP-CVP) = CO × SVR. MAP, mean arterial pressure; CVP, central venous pressure; CO, cardiac output; SVR, systemic vascular resistance. **(C)** The basic 2-element Windkessel model. Elastic artery has specific compliance and behaves as a capacitor. The relation given as: $\text{I}\left(\text{t}\right)=\frac{\text{P}\left(\text{t}\right)}{\text{R}}+\text{C}\frac{\text{dP}\left(\text{t}\right)}{\text{dt}}$ The 3- and 4-element models as a succeeding model are used in recent devices with more accuracy.

Circulatory systems are often compared to electric circuits and are explained by Ohm's law. Ohm's law relates pressure, flow, and resistance by a simple mathematical expression that can be applied to the human circulatory system. In the human body, the amount of electrical current is translated to CO. Electrical resistance correlates to vascular resistance (Figures 1A,B). Consider the following three simple examples as treatment interventions for hypotension after induction of anesthesia: (1) administration of phenylephrine increases SVR, with an increase of BP; (2) administration of dobutamine increases CO, leading to an increase of BP; (3) fluid loading increases CO, with increase of BP. While we recognize BP as an important vital sign, we do not have the tools to directly manipulate BP. It is also not possible to directly measure SVR. By determining BP, CO, and SVR, it is possible to understand which intervention needs to be addressed and which drugs to select and administer. Control of BP is, in essence, hemodynamic management based on CO measurement.

## Monitoring technology

### Non-invasive continuous monitoring

#### Blood pressure

##### Volume clamp

The most popular, noninvasive continuous BP monitor uses finger cuff. Small cuff(s) with photoplethysmogram (PPG) are applied to the fingers. The cuff inflates to cancel out changes in the PPG. The balanced pressure represents the patients' blood pressure at the cuff site. The equipment reconstructs brachial arterial pressure from the finger BP waveform's transformation. It calculates arterial BP using estimated arterial resistance based on the patients' physical characteristics. When a patients' vessel characteristics differ greatly from the installed software, the obtained value may differ greatly from the actual and real value. This technique is still subject to some controversy: while some studies report it as a reliable, others conclude it to be inaccurate. Table 1 summarizes recent studies investigating accuracy of this technique. According to the Association for the Advancement of Medical Instrumentation (AAMI), product standard uses the mean difference in BP measurements between these devices and “gold standard” measurements should be <5 mmHg, with a standard deviation <8 mmHg (3). Alfano et al. compared finger-cuff with a conventional oscillometric method in 40 hemodynamically stable hemodialysis patients (14) and found that the measured values were significantly different between the two methods. This study included an elderly population (65% of whom were over 65 years of age), where vascular calcifications are recognized in up to 88% of the patients. This study looked into patients with diabetes, neuropathy, and increased systolic BP, accounting for its low accuracy measurement. Also, dialysis patients have altered blood vessel characteristics, consistent with concerns derived from the calculation principle. These issues largely overlap with geriatric patient populations. Thus, careful judgment is required regarding the reliability of finger cuff method on elderly patients, especially those with complications as described above and in the cited study. As continuous counter pressure on finger may interfere peripheral blood circulation, these devices set the time limit for continuous use or use two fingers alternately for secured safety.

**Table 1:** Accuracy study of noninvasive continuous blood pressure.

**Study**	**Device**	**Setting**	**No. of subjects**	**Comparison**	**No. of measured values**	**SBP**	**MAP**	**DBP**
Ilies et al. (4)	Finger cuff (CNAP®)	ICU	104	Invasive line (same side radial artery)	11,222	4.3 ± 11.6, 22.8%	−6.1 ± 7.6, 18.4%	−9.4 ± 8.0, 25.6%
Gayat et al. (5)	Finger cuff (CNAP®)	OR (including cardiac surgery)	52	Invasive line (same side radial artery)	5,174	−2 ± 22, 37%	−8 ± 12, 32%	−11 ± 14, 37%
Hahn et al. (6)	Finger cuff (CNAP®)	OR (non-cardiac surgery)	50	Invasive line	237,562	0.9 ± 13.2, NA	−3.1 ± 9.45, NA	−2.8 ± 8.6, NA
Ameloot et al. (7)	Finger cuff (Nexfin®)	ICU	45	Invasive line (femoral artery)	225	8.3 ± 13.8, 22%	−1.8 ± 5.1, 12%	−9.4 ± 6.9, 23%
Vos et al. (8)	Finger cuff (Nexfin®)	OR (non-cardiac surgery)	110	Invasive line (radial artery)	758	NA	2 ± 9, 22%	NA
Hofhuizen et al. (9)	Finger cuff (Nexfin®)	ICU (post cardiac surgery)	20	Invasive line (radial artery)	66	2.7 ± 11.3, NA	4.2 ± 7.0, NA	4.9 ± 6.9, NA
Langwieser et al. (10)	Tonometory (T−line™)	Cardiac ICU	30	Invasive line (radial artery)	7,304	−6 ± 11, 20%	2 ± 6, 17%	4 ± 7, 23%
Meidert et al. (11)	Tonometory (T−line™)	ICU	24	Invasive line (radial artery)	2,993	−3 ± 15, 23%	2 ± 6, 15%	5 ± 7, 22%
Saugel et al. (12)	Tonometory (T−line™)	ICU (medical patient)	22	Invasive line	330	−8 ± 13, NA	0 ± 6, NA	4 ± 6, NA
Findlay et al. (13)	Tonometory (Vasotrac™)	OR (liver transplant)	14	Invasive line (radial artery)	6,468	7.6 ± 13, NA	5.4 ± 10, NA	3.3 ± 8, NA

*SBP, systolic blood pressure; MAP, mean arterial pressure; DBP, diastolic blood pressure. Values of SBP, MAP and DBP are represented as mean difference ± standard deviation in mmHg and percentage error*.

##### Tonometry

Applanation tonometory, on the radial artery, continuously measures the tone calibrated with a conventional arm cuff. Although the first machine was invented in 1963, a major disadvantage of this monitor has been the difficulty in sensor fitting (15). Frequent positioning adjustment and calibration could possibly compensate for errors. When sensor fitting is adequate, a fine arterial pressure waveform is obtained and the system can output both continuous blood pressure and CO values using waveform analysis. Short measurements are widely used for arterial compliance studies. However, long time measurement is currently not common and not commercially distributed.

##### Pulse wave transit time (emerging)

Pulse wave transit time (PWTT) is recognized as a parameter related to hemodynamics, especially SV, BP, and vascular resistance. The device is comprised of a common basic sensor, such as an electrocardiogram and a pulse wave detector on finger (often a pulse oximeter), possibly adding a phonocardiograph. The use of a phonocardiograph can help to provide more precise measurements. In recent years, time resolution, analytical algorithm, and its speed were improved by computer performance and cuff-less BP measurements (16). PWTT is still in development and its accuracy remains poor. Further improvements are needed for its performance, particularly when there is a sudden change in vascular resistance.

#### Cardiac output

##### Bioimpedance and reactance

This method measures the impedance or reactance between a pair of electrodes on the chest wall or on the tracheal tube while applying an imperceptible alternating current that estimates changes in blood volume present in the thorax, particularly in the aorta. Changes in impedance or reactance during one cardiac cycle is considered to reflect stroke volume. This method estimates the stroke volume based on an internal database according to the patients' physical characteristics. Deviation from the database may enhance measurement errors (17). While it is non-invasive, easy to apply and no reported complication associated with an electromagnetic application, it does not detect pure CO. It is also considered to be inaccurate in patients with pulmonary and cardiac pathology. Measurement values will be affected in patients with chronic lung disease, heart, or valvular disease.

##### Ultrasound (echocardiography/transesophageal/transthoracic doppler)

There are roughly two methods to measure flow rate using an ultrasound device: one obtains the SV as the difference of the left ventricular end diastolic volume and the end systolic volume, while the other calculates the SV from the product of a certain cross-sectional area and velocity time integral. Minimally invasive and noninvasive CO monitors are designed with the latter method, which is simple and can reduce operator-dependent discrepancies (18). A variety of dedicated probes are developed for various sites, such as aortic valve, carotid artery, descending aorta, or pulmonary artery. Software can often estimate both the cross-sectional area and proportion of blood flow against SV based on the age and physical characteristics of the patient. This method can be operator dependent and patients' anatomy can sometimes interfere with accurate measurement. The dedicated esophageal probe for CO measurement has a small diameter and low heat emission. Probe insertion and manipulation is rarely associated with oropharyngeal, esophageal, or gastric trauma, but requires appropriate sedation.

##### Pulse transit time

Emerging BP and CO monitoring devices using the relation of SV with PWTT are commercially available and tested (19). Although Pulse Transit Time still needs improvements to increase accuracy, CO can be measured with conventional electrocardiograms and pulse oximeters, and does not require any special sensors or operating techniques. It is considered to be an easy monitor to set up with the added advantage of being noninvasive.

### Minimally invasive continuous CO monitoring

#### Pulse contour analysis

Pulse contour analysis has been investigated and modified since it was first developed. Improved algorithms have been adopted by various commercial devices. Pulse contour analytic CO monitors calculate SV from arterial pressure waveform based on the Windkessel model (Figure 1C) and/or wave reflex phenomenon principle. Pressure waveforms are obtained noninvasively (finger cuff) or minimally invasively (peripheral arterial catheter). In the equation allowing for CO calculation, a constant (κ) reflecting vascular compliance is determined from a preset database that is based on the patients' data (gender, age, height, weight). The databases were developed from a general population, so for patients with complex comorbidities (such as different vascular characteristics, arrhythmia, or valvular heart disease), measurement errors will increase.

Additionally, counter analysis has developed some secondary parameters such as Pulse Pressure Variation (PPV) and Stroke Volume Variation (SVV). These dynamic parameters are used as an index for fluid responsiveness, allowing for appropriate fluid management. The risk of arterial catheter-related infection was reported 1.3% and comparable with 2.7% of central venous catheters (20).

#### Transpulmonary thermodilution

Blood temperature changes are detected by a special arterial cannula which has a thermistor on its tip. Cold fluid boluses are injected through a central venous catheter, which is then sensed in the thermistor tip. CO is calculated from the thermodilution curve according to the Stewart–Hamilton equation. Following this intermittent manual measurement, it continuously calibrates the pulse contour analysis and displays CO. Since calibration is carried out every time the thermodilution is performed, the value is fairly accurate (21). Unlike with pulmonary arterial catheter (PAC), the detected temperature curve is achieved after passing through the pulmonary circulation. The assumption that intra-thoracic blood volume has 1.25 times of global end-diastolic volume allows the system to estimate extravascular lung water without double dilution indicator technique as in the past. Some conditions such as post lung resection or cardiac shunt deteriorate the premise and calculation. The catheter is relatively long and thicker than a regular arterial catheter needing careful insertion to avoid injury.

#### Partial CO_2_ rebreathing

The Fick principle calculates CO with oxygen consumption and arterial and venous oxygen tension. The same principle can be applied to calculate CO_2_ production and blood CO_2_ tension, the indirect Fick method. A dedicated rebreathing loop is connected to the patients' breathing circuit and the system measures CO by calculating carbon dioxide metabolism with partial rebreathing technology. This technology is not affected by vessel anatomical abnormalities or peripheral circulatory insufficiency, as it only needs information from exchange gases. Several validation studies have been published, mainly in the ICU setting (22). This method can only be applied to intubated and mechanically ventilated patients.

Severe lung disease can affect the measurement accuracy due to increased deadspace/tidal volume ratio changing the relationship between PaCO_2_ and PetCO_2_. Acute respiratory distress syndrome is a severe and most common limitation of partial CO_2_-rebreathing. Also, hemoglobin concentration can change the balance between bicarbonate ion and carbon dioxide affecting measurement. It is also not a good method to use in patients with pulmonary hypertension or increased intracranial pressure since they will probably not tolerate CO_2_ retention.

#### Indicator dilution

The Stewart Hamilton equation is behind the basic physics of the indicator dilution method. CO can be measured by an appropriate indicator dye and corresponding detector. Available detectors that do not require blood withdrawal are arterial catheters with a lithium sensor (minimally invasive) and fingertip photometric sensors, which detect indocyanine green (non-invasive). The advantage of products using arterial catheters is that they continuously measure the pulse contour analysis. Repeated measurements with frequent indicator can lead to dye accumulation, resulting in measurement errors and adverse effects. Muscle relaxants (specifically, quaternary ammonium ion) can disturb the lithium ion sensor and rare allergic reactions have been reported with indocyanine green.

A summary of noninvasive and minimally invasive continuous cardiac output monitors available can be found in Table 2.

**Table 2:** Non-minimal invasive continuous cardiac output monitors.

**Basic principle**	**Requirements**	**Advantage**	**Disadvantage**
**NON-INVASIVE**			
Bioimpedance and reactance	Chest wall electrode	Easy installation Continuous measurement	Susceptible to noise
	Dedicated tracheal tube	Continuous measurement	Need Intubation
Ultrasound	TTE probe	Evaluate cardiac preload and motion	Chest wall access Operator's skill
	Transthoracic Doppler probe	Simple and small probe PA based measurement available	Unstable probe direction
Pulse transit time	ECG and pulse oximeter	Calculated from basic monitoring Continuous measurement	Not available in dysrhythmia
**MINIMAL INVASIVE**			
Ultrasound	TEE probe	Evaluate cardiac preload and motion	Esophageal access Operator dependent
	Esophageal Doppler probe	Simple and Small diameter probe GDT Evidence	Esophageal access
Pulse contour analysis	Arterial line	Continuous measurement Evaluate SVV/PPV	Arterial cannulation (covered by noninvasive continuous finger cuff/tonometoric BP technology)
Transpulmonary Thermodilution	Dedicated arterial catheter	Continuous measurement Evaluate preload information (SVV, GEDV, etc)	Central arterial cannulation Manual calibration with cold water injection
Partial CO2 rebreathing	Dedicated breathing circuit	Vascular disease independent	Need intubated and ventilated CO2 loading
Indicator dilution	Dedicated arterial catheter or photometric sensor	Evaluate blood volume	Indicator accumulation/allergy

*TTE, transthoracic echocardiography; TEE, transesophageal echocardiography; ECG, electrocardiogram; GDT, goal directed therapy; SVV, stroke volume variation; PPV, pulse pressure variation; GEDV, global end-diastolic volume*.

## Postoperative outcomes

The appropriate BP values vary from patient to patient, and the “correct” BP may differ depending on the surgery requirements or current situation. A surgical insult may cause the rapid or abrupt change in hemodynamic parameters, making it imperative to continuously monitor BP or other hemodynamic parameters. While controversial, hypotensive anesthesia is practiced with the goal to reduce intraoperative blood loss. This technique requires careful monitoring to avoid dramatic and sudden changes. Patients that have known vascular pathology are also candidates for continuous BP measurement.

Studies showed that sustained intraoperative hypotension is associated with adverse patient outcomes, including increased mortality and organ injury. The duration of hypotension is also an important contributing factor for poor outcomes. Table 3 summarizes several studies that link low BP and adverse outcomes. While there is no definite consensus on the specific degree and duration of hypotension involved, these studies demonstrate the importance of hemodynamic maintenance with individualized considerations. The duration of hypotension was also shown to be an important contributing factor for poor outcomes. Continuous monitoring of hemodynamic parameters would likely reduce the duration of less than desirable BP values and noninvasive, continuous BP monitoring could possibly become the new standard.

**Table 3:** Intraoperative hypotension and adverse outcome.

**Study**	**Study design**	**Type of surgery**	**No. of patients**	**Evaluation of hypotension**	**Outcome measurement**	**Result**	**Remarks**
Sun et al. (28)	Retrospective cohort	Non-cardiac surgery	5,127	MAP < 55, 60, 65 mmHg for 5, 10, 20 min	AKI	MAP < 60 for >10 min associated with AKI	Patients needed invasive BP monitoring
Mascha et al. (29)	Retrospective cohort	Non-cardiac surgery	104,401	Time-weighted average intraoperative MAP	30-day mortality	Intraoperative MAP associated with mortality	Decrease in MAP 80–50 mmHg increased mortality
Monk et al. (30)	Retrospective cohort	Non-cardiac surgery	18,756	Areas under MAP-2SD Absolute BP Percent change from baseline	30-day mortality	Low BP deviation associated with mortality	Absolute BP and % change also associated
Walsh et al. (31)	Retrospective cohort	Non-cardiac surgery	33,330	MAP < 55~75 mmHg for 5, 10, 20 min	AKI and myocardial injury	MAP < 55 associated with AKI and myocardial injury	
Bijker et al. (32)	Case-control	Non-cardiac, non-neurosurgical surgery	294	A priori definition in systolic and mean pressure (40–100 mmHg), Decrease 10–40% of baseline	Ischemic stroke within 10 POD	30% decrease in MAP associated with stroke	Includes 20 CEA patients
Yocum et al. (33)	Cohort	Lumbar spine surgery	45	Absolute BP value	Neuropsychometric performance after 1 day and 1 month	Low minimum MAP associated with low performance	In hypertensive patients
Bijker et al. (34)	Cohort	General and vascular surgery	1,705	A priori definition in SBP and MAP (40–100 mmHg), Decrease 10–40% of baseline	1 year mortality after surgery	Low BP and aging associated with mortality	
Monk et al. (35)	Prospective cohort	Non-cardiac surgery	1,064	SAP < 80 mmHg	1 year mortality	SBP < 80 related to mortality	
Wang NY et al. (36)	Randomized controlled trial	Orthopedic surgery	103	MAP < 80 mmHg	Postoperative delirium at day 2	MAP < 80 mmHg associated to delirium	
Sessler DI et al. (24)	Retrospective	Non-cardiac surgery	24,120	MAP < 75 mmHg	Length of stay and 30-day mortality	Low MAP indicator of mortality	

*BP, blood pressure; MAP, mean arterial pressure; SBP, systolic blood pressure; CEA, carotid endarterectomy; AKI, acute kidney injury; SD, standard deviation*.

The perioperative hemodynamic management of surgical patients extends beyond cardiovascular complications. Delayed recovery of cognition, whether delirium (an acute attentional deficit which waxes and wanes), or the long-lasting phenotype termed postoperative cognitive decline (POCD), has been linked to intraoperative blood pressure fluctuations (23) or maintained hypotension in the intraoperative period (24). The use of vasopressors during surgery and/or postoperative hypertension is associated with new-onset dementia after surgery (25). With more than 46 million Americans over the age of 65, postoperative delirium is a major public health issue with an projected annual cost of over $150 billion. It is estimated that 30–40% of delirium cases might be preventable (26). Prevention and optimization is the most effective strategy for minimizing neurologic injury. Hemodynamic monitoring using minimally invasive and noninvasive monitors can optimize the cognitive recovery and perioperative experience of surgical population. This might lead to improve neurologic outcomes, decrease hospital length of stay, reduce the amount of postoperative mechanical ventilation, lessen ICU length of stay, cut back healthcare costs in general, and patients' functional decline.

## Cardiac output

### In the operating room

Numerical, target-oriented volume and inotropic management based on hemodynamic measurement is crucial for a rapid recovery. It is increasingly accepted that the traditional fixed volume therapy should be abandoned and the administration of fluids to achieve a certain volume (goal-directed fluid therapy) improves outcomes. In addition to pressure measurement, hemodynamic parameters such as SV need to be calculated (27) and minimally invasive devices can be used, for example in high risk ERAS cases.

### In the intensive care unit (ICU)

The International Guidelines for Management of Severe Sepsis and Septic Shock brought further attention to the need for hemodynamic assessment in critically ill patients (37). Management in the ICU is based on a detailed assessment, which includes infusion loading, diuretics, dialysis, cardiovascular drugs, ventilator setting, rehabilitation care, and timing. Along with patient recovery, removing unnecessary invasive monitors, and their replacement with minimally invasive techniques can reduce mechanical and infectious complications, facilitating early mobilization and recovery. Many patients have an arterial line for frequent blood sampling in ICU. Pulse contour analysis monitor is therefore an option since CO and other parameters can be obtained without inserting an additional catheter.

## Discussion

No single monitor is able to comprehensively identify the spectrum of pathophysiologic changes for high risk patients, despite various commercially available devices with a range of differing measurement principles.

Understanding the measurement principles behind minimally invasive and noninvasive techniques can facilitate accurate evaluation of patients' hemodynamic status, even taking into consideration a possible measurement mismatch. When choosing and applying these monitors, it is important to clarify the purpose for monitoring and how to correctly employ the obtained parameters. The development of minimally invasive and noninvasive devices derives from the need to reduce complications from invasive tools. The application of two complementary devices, with different background principles, might even be an alternative to an invasive technology.

In order to improve patient outcomes, monitoring itself should not be the goal. Monitoring principles need to be understood to guide therapy and decision making. New techniques have led to the development of new hemodynamic parameters. Dynamic parameters such as SVV and PPV are now widely recognized as important signs that can be used to guide fluid management. SVV has been shown to be a valid measure of fluid responsiveness (38, 39) and many different technologies are available for measuring SVV at the bedside (40). An estimate of both SVV and PPV is displayed in real time by the PiCCO plus system (Pulsion Medical Systems AG) (38, 41) as well as by the LiDCO system. The pulse contour method measures SVV through a femoral catheter (transcardiopulmonary thermodilution) (42, 43). Another device that measures SVV, the FloTrac/Vigileo system (Edwards Lifesciences LLC), requires standard arterial access and is considered minimally invasive and easy to use (44). In an RCT in patients who had undergone elective cardiac surgery (*N* = 40), SVVs assessed using the FloTrac/Vigileo and the PiCCOplus systems performed similarly in predicting fluid responsiveness (42). Today many studies have demonstrated the ability of this algorithm to predict fluid responsiveness in the operating room. It is also possible to assess surrogates for SVV and PPV non-invasively. Attached noninvasively to a finger (45), the pulse oximeter probe can be used to detect changes in blood volume at the site of measurement (46) and respiratory variations in the pulse oximeter plethysmographic waveform amplitude (ΔPOP) have been shown to be related to PPV and SVV (47). This index is also sensitive to changes in preload (48), and can predict fluid responsiveness in mechanically ventilated patients (46, 49–52). The Pleth Variability Index (PVI) is a clinical algorithm that allows for noninvasive, automated, continuous calculation of ΔPOP using a pulse oximeter in mechanically-ventilated patients during general anesthesia (40, 45, 53). PVI is calculated as the dynamic changes in perfusion index (PI)—the ratio of non-pulsatile to pulsatile blood flow through the peripheral capillary bed—occurring during a complete respiratory cycle (40, 54). PVI has been shown to predict fluid responsiveness with good sensitivity and specificity: in mechanically ventilated patients (45). Today, SVV is also available non-invasively using Bioreactance (NICOM, Cheetah) and technologies based on non-invasive blood pressure monitoring (Clearsight, CNAP devices). It is possible that the future will bring us even better indicators derived from advanced method and analysis. Although the comparative examination on the accuracy of the new equipment will require intensive studies, we can wait in anticipation of these new technologies.

The assessment of hemodynamics allow for a customized approach to patient management, one in which treatment decisions are being guided by more precise, multimodal and technologically sophisticated monitoring of physiological variables. Monitoring equipment that can provide precise hemodynamic information without the complications and complexity of invasive techniques can facilitate individualized hemodynamic management and lead to improved outcomes and many other positive contributions to the field.

## Author contributions

TY, SV, and MC are responsible for manuscript research, writing, editing, and review. YG is responsible for manuscript review.

### Conflict of interest statement

MC is a founder of Sironis and holds a patent on closed-loop hemodynamic optimization licensed to Edwards Lifesciences. The other authors declare that the research was conducted in the absence of any commercial or financial relationships that could be construed as a potential conflict of interest.
